# Combination therapy with pazopanib and tivantinib modulates VEGF and c-MET levels in refractory advanced solid tumors

**DOI:** 10.1007/s10637-021-01138-x

**Published:** 2021-06-28

**Authors:** Shivaani Kummar, Apurva K. Srivastava, Tony Navas, Fabiola Cecchi, Young H. Lee, Donald P. Bottaro, Sook Ryun Park, Khanh T. Do, Woondong Jeong, Barry C. Johnson, Andrea R. Voth, Larry Rubinstein, John J. Wright, Ralph E. Parchment, James H. Doroshow, Alice P. Chen

**Affiliations:** 1 Division of Cancer Treatment and Diagnosis, National Cancer Institute, Bethesda, MD 20892 USA; 2 Clinical Pharmacodynamics Biomarkers Program, Applied/Developmental Research Directorate, Frederick National Laboratory for Cancer Research, Frederick, MD 21702 USA; 3 Urologic Oncology Branch, NCI, Bethesda, NIH 20892 USA; 4 Biostatistics Branch, National Cancer Institute, Bethesda, MD 20892 USA; 5 Center for Cancer Research, National Cancer Institute, Bethesda, MD 20892 USA; 6 Division of Hematology and Medical Oncology, Knight Cancer Institute, Oregon Health & Science University, Portland, OR 97239 USA; 7 Pfizer Inc, Groton, CT 06340 USA; 8 AstraZeneca, Inc, Gaithersburg, MD 20878 USA; 9 Altimmune, Inc, Gaithersburg, MD 20878 USA; 10 Department of Oncology, University of Ulsan College of Medicine, Seoul, 138-736 South Korea; 11 Moderna Therapeutics, Inc, Cambridge, MA 02139 USA; 12 Millennium Physicians, Tomball, TX 77375 USA

**Keywords:** Biomarkers, Pharmacodynamics, Pazopanib, Tivantinib, Angiogenesis

## Abstract

The vascular endothelial growth factor (VEGF)/VEGFR and hepatocyte growth factor (HGF)/c-MET signaling pathways act synergistically to promote angiogenesis. Studies indicate VEGF inhibition leads to increased levels of phosphorylated c-MET, bypassing VEGF-mediated angiogenesis and leading to chemoresistance. We conducted a phase 1 clinical trial with 32 patients with refractory solid tumors to evaluate the safety, pharmacokinetics, and pharmacodynamics of combinations of VEGF-targeting pazopanib and the putative c-MET inhibitor ARQ197 (tivantinib) at 5 dose levels (DLs). Patients either took pazopanib and tivantinib from treatment initiation (escalation phase) or pazopanib alone for 7 days, with paired tumor sampling, prior to starting combination treatment (expansion phase). Hypertension was the most common adverse event. No more than 1 dose limiting toxicity (DLT) occurred at any DL, so the maximum tolerated dose (MTD) was not determined; DL5 (800 mg pazopanib daily and 360 mg tivantinib BID) was used during the expansion phase. Twenty of 31 evaluable patients achieved stable disease lasting up to 22 cycles. Circulating VEGF, VEGFR2, HGF, and c-MET levels were assessed, and only VEGF levels increased. Tumor c-MET levels (total and phosphorylated) were determined in paired biopsies before and after 7 days of pazopanib treatment. Total intact c-MET decreased in 6 of 7 biopsy pairs, in contrast to previously reported c-MET elevation in response to VEGF inhibition. These results are discussed in the context of our previously reported analysis of epithelial-mesenchymal transition in these tumors.

**Supplementary information:**

The online version contains supplementary material available at 10.1007/s10637-021-01138-x.

## Introduction

Angiogenesis is associated with tumor growth and metastasis. Hypoxic conditions in rapidly growing tumors induce pro-angiogenic factors such as vascular endothelial growth factor (VEGF) and the receptor tyrosine kinase c-MET via hypoxia-inducible factor (HIF)-1α [1, 2]. High levels of VEGF receptor (VEGFR), VEGF, c-MET, and the c-MET ligand hepatocyte growth factor (HGF) are associated with poor prognosis and tumor aggressiveness [3–6]. The VEGF and HGF signaling pathways synergistically mediate angiogenesis and transcriptional activation following simultaneous exposure to both ligands [7–9].

Upregulation of c-MET levels in response to anti-VEGF antibody treatment indicates interplay between the two pathways in preclinical models [10]. Itatani and colleagues reviewed the changes observed in other angiogenic pathways that occur during anti-VEGF treatment [11], including increased c-MET phosphorylation in mouse glioblastoma models [12]. Therefore, combination therapies targeting both HGF/c-MET and VEGF/VEGFR signaling may be useful in overcoming resistance to long-term anti-angiogenic treatment [10, 13].

Under hypoxic conditions in preclinical studies, HIF-1α also regulates a range of epithelial-mesenchymal transition (EMT)-inducing factors, including Snail1 [14–16], TCF3 and ZFHX1A/B [17], SIP1 [15], LOX [18], and TWIST [19]. This indicates that hypoxia associated with anti-angiogenic therapy may trigger EMT in human carcinomas. Non-angiogenic autocrine VEGF signaling also appears to contribute to aggressive behavior in tumor cells by enhancing nuclear localization of Snail1 [20, 21], thus driving EMT [16], but it is unclear how this mechanism is affected by anti-angiogenic therapy.

Pazopanib is a potent, FDA-approved tyrosine kinase inhibitor [22] that targets VEGFR1, VEGFR2, and VEGFR3, as well as PDGFR-α, PDGFR-β, and c-KIT, which are all components of angiogenesis-associated signaling pathways aberrantly activated in carcinogenesis [23–25]. Pazopanib therapy is associated with inter- and intra-patient variability in its pharmacokinetics [26]. Pazopanib is both a substrate and inhibitor of CYP3A4, although no association between CYP3A4 polymorphisms and differential metabolism has been demonstrated [27–30]. Tivantinib, initially reported to act as a c-MET inhibitor, has been found to exert antitumor activity through the inhibition of microtubule assembly [31, 32]. CYP2C19 polymorphisms have been shown to cause approximately twofold differences in tivantinib’s AUC between poor metabolizers and extensive metabolizers [33].

This phase 1 study was designed to test whether the combination of pazopanib and tivantinib could inhibit angiogenesis by inhibiting two distinct signaling pathways. This objective was complicated by off-target effects of tivantinib reported after the escalation phase had begun. Therefore, the study was amended so that the pharmacodynamic consequences of single-agent pazopanib treatment could be explored through quantitation of total and phosphorylated c-MET in paired tumor biopsies; longitudinal analyses of plasma levels of circulating HGF, c-MET, VEGFR2, and VEGF were examined during pazopanib + tivantinib combination treatment. Safety, pharmacokinetics, and clinical outcomes were also assessed.

## Patients and methods

### Eligibility criteria

Patients ≥ 18 years old were eligible if they had a pathologically confirmed solid tumor that had progressed following at least one line of prior therapy or had no acceptable standard therapies. Patients enrolled in the expansion cohort were required to have malignancies where prior reports suggested a potential oncogenic role for c-MET signaling [34], to have disease amenable to biopsy, and to be willing to undergo pre-and post-treatment biopsies. All previous anticancer therapies should have been completed at least 4 weeks prior to enrollment. Patients were required to have an Eastern Cooperative Oncology Group (ECOG) performance status ≤ 1 and adequate organ and marrow function defined as an absolute neutrophil count ≥ 1,500/μL, platelets ≥ 100,000/μL, total bilirubin ≤ 1.5 × the upper limit of normal (ULN), aspartate aminotransferase and/or alanine aminotransferase < 2.5 × ULN, and creatinine < 1.5 × ULN, and urine protein/creatinine ratio < 1 or 24-h urine protein < 1 g. Well-controlled blood pressure (BP), defined as a BP not greater than 140 mm Hg (systolic) and 90 mm Hg (diastolic) was also required for eligibility. Initiation or adjustment of BP medication was permitted prior to study entry if the average of 3 BP readings at a visit prior to enrollment was more than 140/90 mm Hg. Patients were excluded if they had an uncontrolled intercurrent illness, were pregnant or lactating, or had received treatment for brain metastases within the past 3 months. Prior c-MET inhibitor therapy or anti-angiogenic therapy was allowed in the escalation cohort but not in the expansion cohort; prior anti-angiogenic therapy should have been completed at least 3 months prior to enrollment. This trial was approved by the NCI Institutional Review Board; protocol design and conduct followed all applicable regulations, guidances, and local policies. Written informed consent was obtained from all participants.

### Study design

This was an open-label phase 1 study of pazopanib and tivantinib in patients with advanced malignancies (NCT01468922). The Division of Cancer Treatment and Diagnosis, NCI, supplied pazopanib and tivantinib under Collaborative Research and Development Agreements with GlaxoSmithKline and Arqule, respectively. Pazopanib was administered orally daily without food (at least 1 h before or 2 h after eating); tivantinib was administered orally with food twice daily approximately every 12 h. Five dose levels (DLs) were evaluated using a standard 3 + 3 dose escalation design. Higher DLs were not opened until the last patient in the previous cohort had completed one cycle. The maximum tolerated dose (MTD) was defined as the highest dose level at which no more than 1 of 6 patients experienced a dose-limiting toxicity (DLT). Once the escalation phase was complete, patients enrolled on the expansion phase were treated with pazopanib alone (at the pazopanib MTD) for one week before beginning combination treatment on day 8.

### Safety assessments

Adverse events were graded according to NCI Common Toxicity Criteria version 3.0. Patients were considered evaluable for toxicity for the purpose of dose escalation decisions if they either 1) experienced a DLT or 2) received at least 80% of the planned 28-day course of treatment in one cycle of therapy and were followed for one full cycle. DLT was defined as an adverse event that occurred in the first cycle, was felt to be related to the study drugs, and fulfilled one of the following criteria: grade 3 or greater non-hematologic toxicity (except grade 3 nausea/vomiting and diarrhea without maximal symptomatic treatment or grade 3 creatinine and electrolyte abnormalities that corrected to grade 1 or baseline within 24 h); grade 4 neutropenia lasting ≥ 3 days or febrile neutropenia; grade 4 thrombocytopenia; QTc prolongation to ≥ 500 ms on at least two separate EKGs; a ≥ 2-week delay in starting the next cycle due to toxicity; a significant elevation in liver test, defined as AST or ALT > 8 × ULN or AST or ALT > 3 × ULN with concurrent bilirubin elevation > 2 × ULN; grade ≥ 3 bleeding in any site or grade ≥ 2 pulmonary hemorrhage; or grade 4 venous thrombosis, pulmonary embolism, or any grade of arterial thrombosis. Any degree of anemia, leukopenia in the absence of neutropenia, or lymphopenia were not considered dose limiting. Doses of study drugs were held for DLTs, and toxicities had to resolve to grade ≤ 2 before receiving further dosing on study at the next lowest dose level. Treatment could be delayed for a maximum of 2 weeks to allow resolution of toxicities. If toxicities did not resolve as defined above, patients were taken off study treatment.

### Pharmacokinetics

Blood samples for PK analysis were collected on cycle 1 day 1 (hereafter, cycle x day y is denoted as CxDy) before drug administration and 1, 2, 4, 6, and 12 h after administration of pazopanib (the 12-h time point was collected before the second dose of tivantinib), and at C1D15 and C2D1 before drug administration and 1, 2, 4, and 6 h after administration of pazopanib. All blood samples were centrifuged at 1500 × g at 4 °C for 10 min, and the resulting plasma was stored at ≤ -70 °C until analysis. Plasma pazopanib and tivantinib levels were analyzed by Non-Compartmental Analysis using Phoenix/WinNonLin V6.4.

### Soluble biomarkers

Blood samples for analysis of soluble markers were collected on C1D1 pre-dose and 2, 4, and 6 h post-pazopanib dose, and on C1D15 and C2D1 prior to drug administration. Soluble c-MET ectodomain and HGF protein content in plasma were measured using a two-site electrochemiluminescent immunoassay developed for use with a Meso Scale Discovery Sector S600 plate reader as described previously [35]. Antibodies for capture (c-MET, BAF-358; HGF, MAB-694) and detection (c-MET, AF-276; HGF, AF-294) were from R&D Systems (Minneapolis, MN). Detection antibodies were tagged with a ruthenium chelate (Sulfo-Tag, Meso Scale Discovery, Gaithersburg, MD) following the manufacturer’s instructions. Purified recombinant proteins (c-MET ectodomain-Ig fusion protein [358-MT], HGF [294-HG], R&D Systems) were used to construct standard curves from which plasma content values were derived. Assays for VEGFA (K151UVK) and soluble VEGFR2 (K151BOC) were obtained from Meso Scale Discovery and performed according to the manufacturer’s instructions.

### Total and phosphorylated c-MET in tumor biopsies

Paired tumor biopsies (18-gauge core needle biopsies, 2 cores) were obtained from one patient in the dose escalation phase and all patients on the expansion cohort at baseline (pre-treatment) and on C1D8 after pazopanib treatment, but before the first dose of tivantinib. Biopsies were placed in prechilled cryogenic vials, frozen within 2 min of collection, and stored ≤ -80 °C. Total and phosphorylated c-MET levels were determined using an immunoassay as previously described [35]. Multiplex immunofluorescence was performed on formalin-fixed, paraffin-embedded (FFPE) tumor tissues; 10 µg/mL mouse monoclonal DIG-conjugated anti-pY1235-MET clone 23111 [35], 2.5 µg/mL AF488-conjugated rabbit monoclonal anti-MET (clone D1C2, Cell Signaling Technology, #8494), and 1 µg/mL antibody to Na^+^/K^+^ ATPase directly conjugated to AF647 (clone EP1845Y, ab76020) were used as primary antibodies, followed by 10 µg/mL mouse anti-DIG-Cy3 (Jackson Immunoresearch, West Grove, PA) as a secondary antibody. Image acquisition and analysis were performed on an Aperio Slide Scanner and Definiens Software (Tissue Studio and Developer) [36].

## Results

### Demographics

Thirty-two patients with solid tumors were enrolled in the study (Supplementary Table S1). One male patient did not meet eligibility criteria and was released from the study; thus, 31 patients completed at least one cycle on the study. The median age was 54 years. Treated patients had received a median of 4 prior therapies (range: 0–13).

### Safety

Adverse events (AEs) observed at all DLs are listed in Supplementary Table S2. One patient experienced grade 3 lymphopenia at DL1; all other grade 3 or 4 AEs occurred at DL4 or DL5. All grade 4 AEs occurred at DL5 and were related to immune suppression, including febrile neutropenia (*n* = 2), leukopenia (*n* = 2), lymphopenia (*n* = 1), neutropenia (*n* = 2), and sepsis (*n* = 2). Grade 3 AEs included those related to immune suppression, elevated liver enzymes, and hypertension. Hypertension was the most common AE (grade 2: *n* = 15 across all DLs; grade 3: *n* = 3, only at DL4-5). The MTD was not determined, as no more than 1 DLT occurred at any DL. Therefore, DL5 (800 mg of pazopanib once daily and 360 mg of tivantinib twice daily) was used for the expansion phase.

**Fig. 1:**
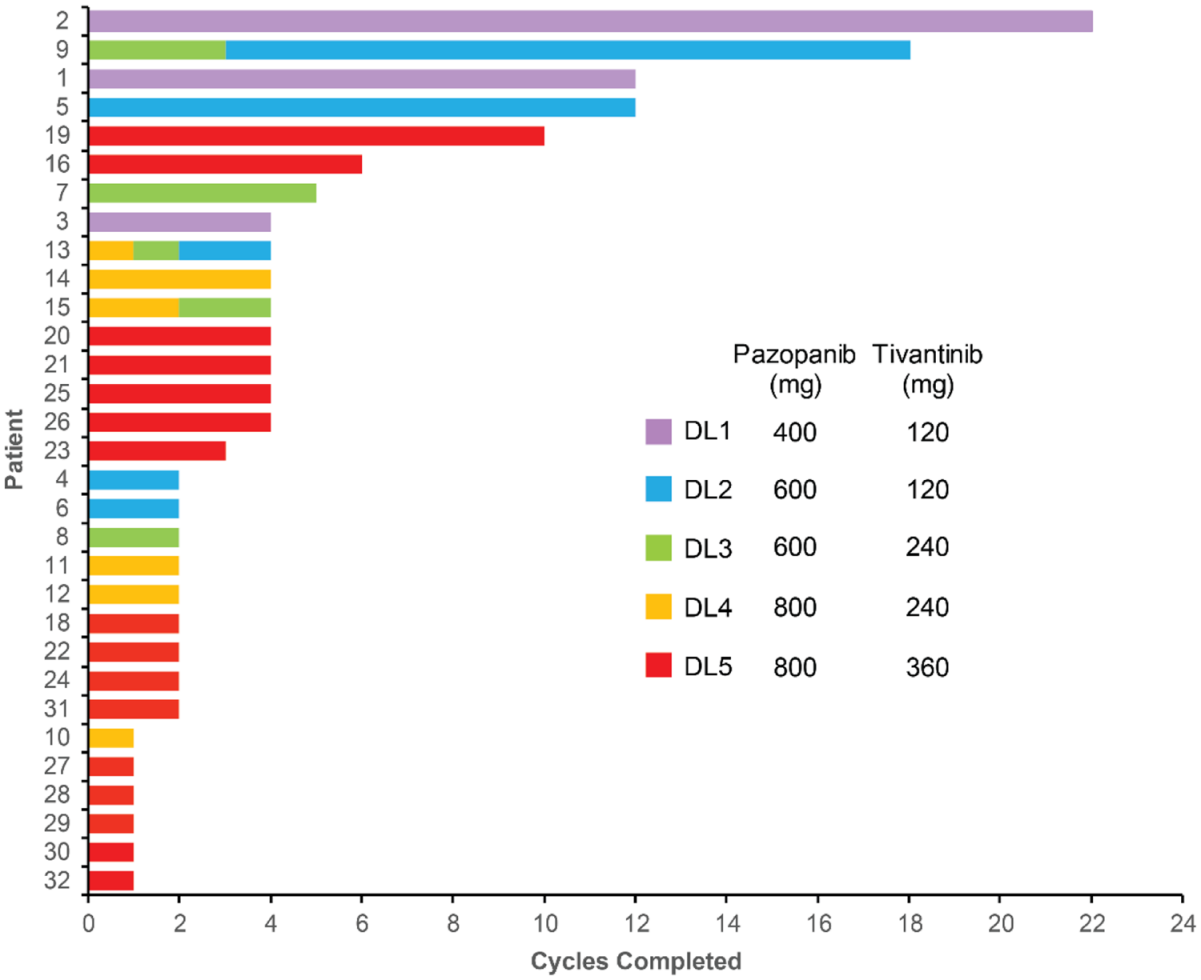
Cycles completed by each patient. Participants 9, 13, and 15 required dose reductions and completed additional cycles at lower dose levels as indicated by multi-colored bars

Three patients (9, 13, and 15) required dose reductions. One of these patients (patient 9) dropped from DL3 to DL2 due to hypertension after cycle 3, then remained on DL2 through cycle 18 (Fig. 1). The other dose reductions (patients 13 and 15) were due to blood count suppression (leukopenia, lymphopenia, and neutropenia). Patient 15 dropped from DL4 to DL3 after cycle 2, staying at DL3 through cycle 4; patient 13 started at DL4 and had dose reductions after cycles 1 and 2, ultimately staying at DL2 through cycle 4.

### Clinical outcomes

Patients were treated with the pazopanib + tivantinib combination for 1 to 22 cycles (Fig. 1). Five patients (patients 1, 2, 5, 9, and 19) had a best response of stable disease for ≥ 10 cycles; an additional 10 patients had ≥ 4 cycles of stable disease. Of the five patients with stable disease for ≥ 10 cycles, three (patients 1, 2, and 5) started at DL1 or DL2; another patient (patient 9) started at DL3 and was changed to DL2 after 3 cycles and remained at DL2 through cycle 18. Only patient 19 started at DL3 or higher and remained at the initial dose with stable disease for ≥ 10 cycles. No patient exhibited an objective response to treatment. Enrolled patients presented with many different tumor types (Supplementary Table S3). Low numbers of each tumor type precluded any interpretation of responses based on histology; however, it is noted that the two longest courses of treatment (22 and 18 cycles, patients 2 and 9, respectively) were in the only two patients with alveolar soft part sarcoma (ASPS), a disease characterized by increased expression of MET [37].

### Pharmacokinetics

There was no accumulation of either agent between days 1 and 15 (Supplementary Tables S4 [pazopanib] and S5 [tivantinib]). AUC values for pazopanib at 600 mg or 800 mg doses (DL2 through DL5) did not increase between these time points (*P* = 0.54 and 0.27 at 600 mg and 800 mg, respectively, Supplementary Table S4). There was no significant increase in pazopanib exposure with dose escalation from 600 to 800 mg (*P* = 0.43 and 0.60 on day 1 and day 15, respectively). Given the narrow dose escalation range, any resulting changes in pazopanib exposure may have been too subtle for detection. Additionally, the sampling schedule did not allow full characterization of the elimination kinetics of pazopanib or tivantinib.

Mean AUC values indicate that tivantinib exposure did not increase between 120 mg doses (DL1 and DL2) and 240 mg doses (DL3 and DL4) on days 1 and 15 (*P* = 0.33 and 0.52, respectively, Supplementary Table S5). However, tivantinib AUC values were significantly lower on day 15 than on day 1 at the 360 mg dose (DL 5, 11,497 ± 8041 h∙ng/mL versus 3083 ± 1740 h∙ng/mL, *P* = 0.031); C_max_ did not change significantly between these time points (1589 ± 978 ng/mL on day 1 versus 934 ± 387 ng/mL on day 15, *P* = 0.16).

### Circulating VEGF levels following combination treatment

**Fig. 2:**
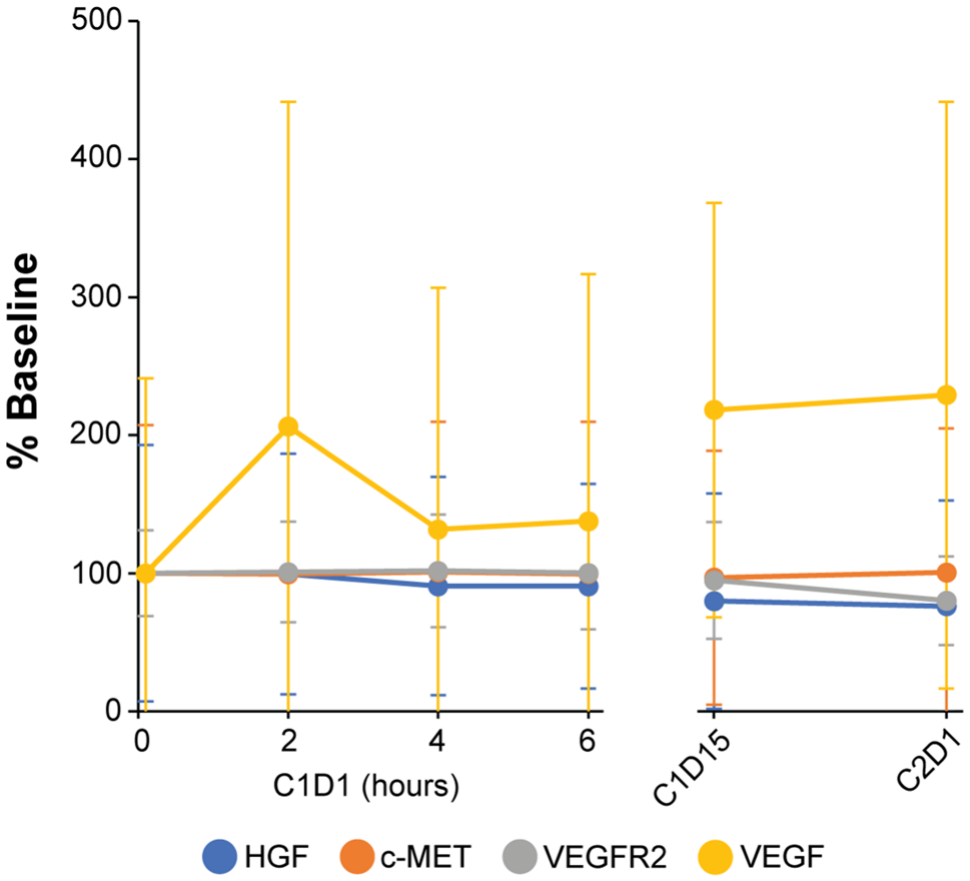
Circulating HGF/c-MET/VEGFR2/VEGF levels on C1D1, C1D15, and C2D1, shown as the percent of each biomarker’s pre-treatment level. Mean normalized values and standard deviations are shown for patients 1 through 22 who received both pazopanib and tivantinib from day 1

VEGF levels increased across all DLs by C2D1 (Fig. 2) in escalation phase patients (patients 1–22). Due to wide distributions, differences from baseline did not achieve statistical significance at any time point by unpaired Student’s t-test (assuming unequal variance), although VEGF levels on C1D15 and C2D1 were closest to doing so (*P* = 0.073 and 0.060, respectively). Circulating c-MET, VEGFR, and HGF concentrations showed little change at any time point (Fig. 2).

### Tumor c-MET levels following pazopanib treatment

**Fig. 3:**
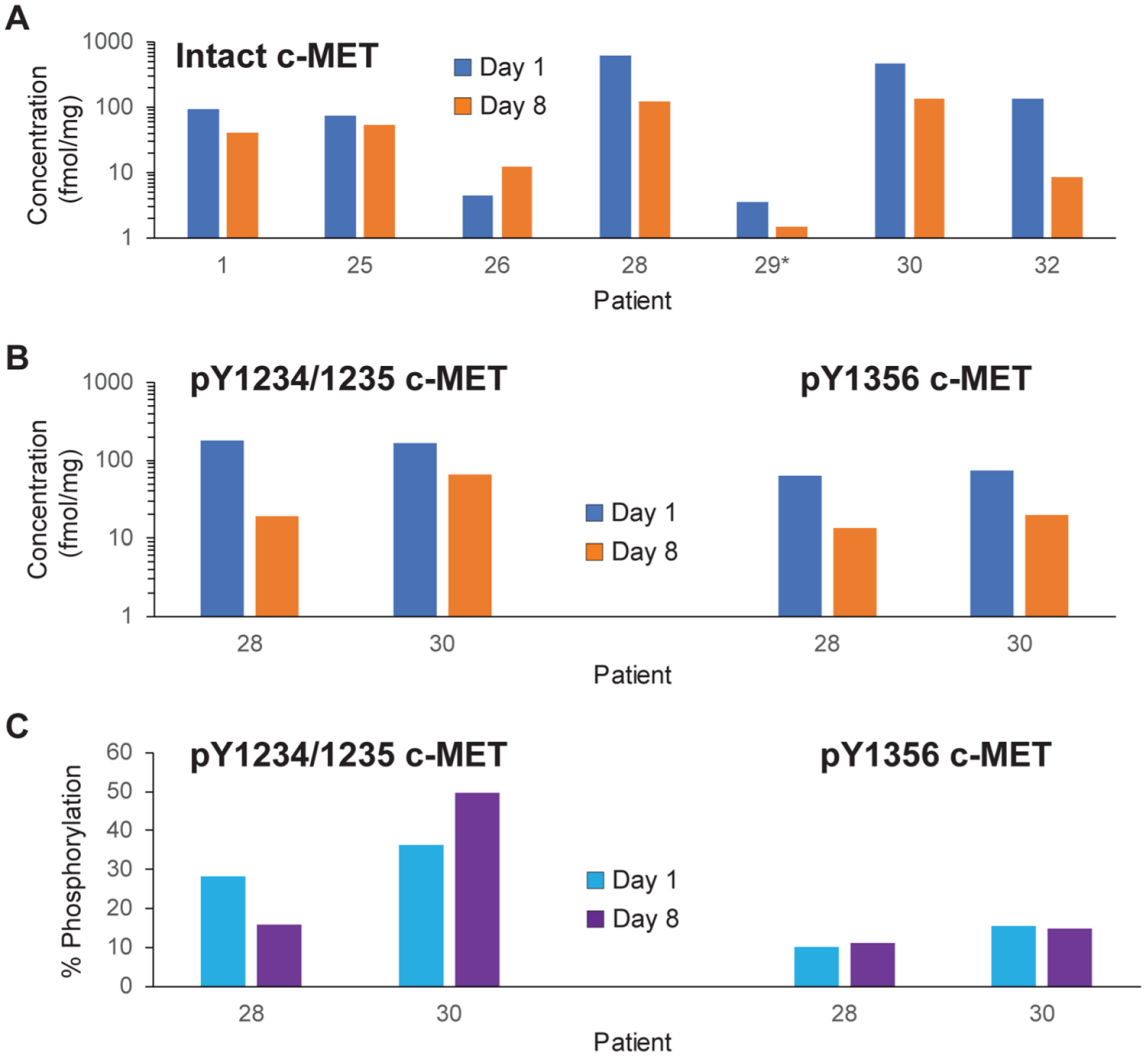
Total and phosphorylated c-MET levels in tumors. (**A**) Levels of intact c-MET were determined in tumor biopsies from 7 patients on C1D1 and C1D8, after pazopanib administration. The asterisk for patient 29 denotes that the intact c-MET level on C1D8 was below the lower limit of quantitation (LLQ, 1.50 fmol/mg). (**B**) Site-specific phosphorylation was quantifiable on both days for 2 patients (levels above LLQ on both days). Phosphorylated c-MET levels for patients 1, 25, 26, and 29 were below the LLQ (1.50 to 7.81 fmol/mg) at both time points. (**C**) Percent phosphorylation at both phosphorylation sites was determined for patients 28 (ovarian cancer) and 30 (esophageal cancer)

**Fig. 4:**
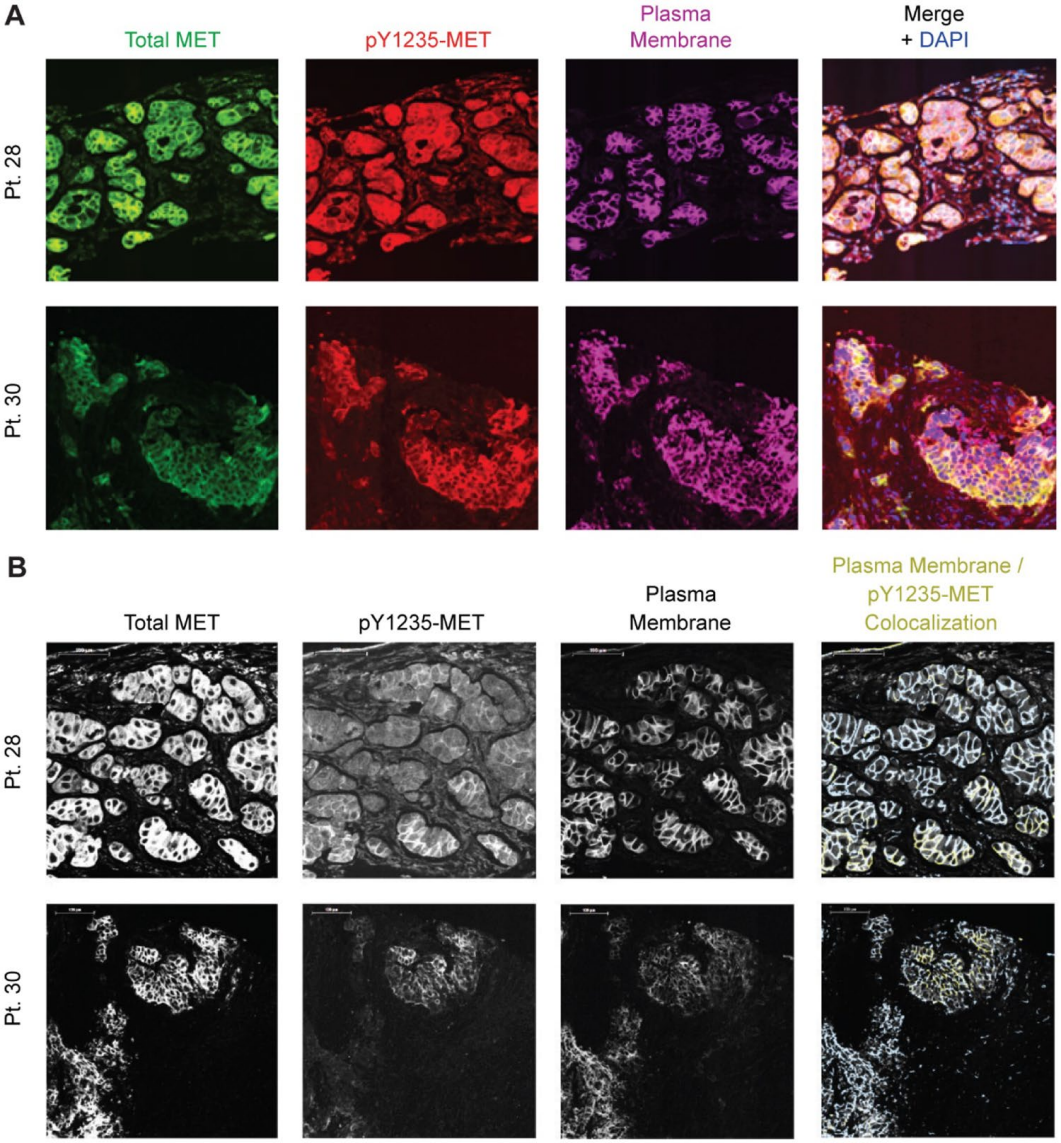
Total MET and pY1235-MET localization. (**A**) Patient tumors were stained for total MET (green), pY1235-MET (red), plasma membrane marker Na^+^/K^+^-ATPase (magenta), and DAPI (blue). (**B**) High-magnification fluorescence images showing staining for total MET, pY1235-MET, and Na^+^/K^+^-ATPase, and masked images showing regions of Na^+^/K^+^-ATPase colocalized with pY1235-MET (gold) within the plasma membrane mask (light blue)

Intact and phosphorylated c-MET were measured in paired biopsy samples from 9 patients (1 at DL1, 8 at DL5) before treatment and after 7 days of treatment with pazopanib; intact c-MET signals from 7 of these patients were above the lower limit of quantitation (LLQ). Two patients’ biopsies (patient 28 with ovarian carcinoma and patient 30 with esophageal carcinoma) had signal above the LLQ for c-MET phosphorylated at either Y1234/1235 or pY1356 in both biopsies. Absolute levels of intact c-MET decreased in 6 of 7 patients’ tumors between C1D1 and C1D8 (Fig. 3A); pY1234/1235-MET and pY1356-MET decreased in biopsies from patients 28 and 30 (Fig. 3B). The percent of intact c-MET phosphorylated at Y1356 was consistent in both patients at both time points, while the percentage of c-MET phosphorylated at Y1234/1235 decreased in patient 28 and increased in patient 30 (Fig. 3C). Low levels of total c-MET in biopsies from other patients limited quantitation of phospho-MET in these samples. Immunofluorescence staining of FFPE tissue was performed to evaluate whether MET and phospho-MET signals derived from tumor or stroma (Fig. 4). Phospho-MET analyses in these experiments were performed with an antibody specific to pY1235-MET rather than the pY1234/1235-MET antibody used in the analyses of lysed biopsy cores, and the phospho-MET signal was unambiguously traced to the tumor cells.

## Discussion

This trial evaluated the pharmacodynamic effects of pazopanib alone and the pazopanib + tivantinib combination. Pharmacokinetics and circulating VEGF, VEGFR2, HGF, and MET levels were evaluated in patients taking the combination of pazopanib and tivantinib; tumor pharmacodynamic responses were assessed in patients receiving only pazopanib for 7 days prior to initiating combination therapy. Safety and clinical outcomes were assessed in all patients.

Our results are consistent with previously reported variability in pazopanib pharmacokinetics [26]. This variability has been linked to pH-dependent absorption affected by the timing of doses relative to meals and its high protein binding affinity (greater than 99.9% in some patients), leaving the small unbound fraction susceptible to large relative changes due to interpatient variability [38–40]. A steady-state trough concentration threshold of 20.5 µg/mL has been associated with improved progression-free survival and higher prevalence of adverse events, including hypertension [41, 42]. While many patients achieved this threshold (Supplementary Table S4), pazopanib did not accumulate in plasma between C1D1 and C1D15.

Analysis of circulating biomarker levels showed consistent and sustained increases in VEGF levels after treatment, while HGF, c-MET, and VEGFR2 levels remained near baseline values (Fig. 2). Pazopanib inhibits all three VEGFR isoforms [43], but only VEGFR2 was measured as it has been identified as the primary VEGF receptor for endothelial proliferation [44]. The possibility remains that levels of other VEGFR isoforms fluctuated in response to pazopanib or to changing levels of the circulating ligand. Similar fluctuations were not observed with circulating c-MET, while intact c-MET levels in paired tumor biopsies did not increase as in previous studies [10, 13]. Activation of c-MET transcription has been reported in response to hypoxic tumor conditions in cultured cell lines derived from tumor samples [45]. Increased percent c-MET phosphorylation at either Y1234/1235 or Y1356 was observed in one out of two patients with detectable phospho-signal in this study (patient 30, esophageal carcinoma). Decreased total c-MET levels in 6 of 7 patients suggest another compensatory mechanism(s) may be involved in tumor responses.

Detailed analyses of how anti-angiogenic treatments affect epithelial-mesenchymal phenotype are necessary both for dissecting the interplay between EMT and angiogenesis in promoting cancer progression and for determining the best clinical treatment course based on tumor EMT profiling. To this end, we developed a tool for measuring the epithelial-mesenchymal phenotype of tumors by simultaneously quantifying the epithelial marker E-cadherin (E) and the mesenchymal marker vimentin (V), called the EMT-IFA [46]. Paired biopsies from seven evaluable patients revealed that 4 patients (patients 25, 26, 28, and 29) had a significant shift toward a mesenchymal phenotype following 7 days of pazopanib treatment, as assessed by the log10(V/E) ratio and relative changes in the total numbers of tumor cells with E + -only, V + -only, and mixed V + E + phenotypes [46]. Total intact c-MET, pY1234/1235-MET and pY1356-MET decreased from day 1 to day 8 in patients 28 (ovarian carcinoma) and 30 (esophageal carcinoma). The percentage of c-MET phosphorylated at Y1356 remained consistent in both patients, while the percent of c-MET phosphorylated at Y1234/1235 decreased in patient 28 and increased in patient 30. Biopsies from patient 28 showed a shift toward a mesenchymal phenotype by EMT-IFA analysis; no such shift was observed in biopsies from patient 30 [46]. Three patients’ tumors (25, 26, and 29, with renal cell carcinoma, unspecified carcinoma, and non-small cell lung carcinoma, respectively) showed an EMT shift without having quantifiable levels of phospho-MET. Total intact c-MET levels decreased in patients 25 and 29, while intact c-MET increased in patient 26. Tumors from patients 23 (chondrosarcoma) and 24 (myoepithelial adenocarcinoma of the parotid gland) did not show evidence of EMT; intact c-MET levels in these tumors were below the LLQ. A relationship between MET activation and epithelial-mesenchymal phenotype changes could not be identified from these data.

No patient evaluated for EMT status remained on study for longer than 4 cycles (Fig. 1). Patients whose biopsies showed evidence of a mesenchymal shift (patients 25, 26, 28, and 29) progressed after 2.5 ± 1.7 cycles (range: 1–4 cycles). Biopsies from patients 23, 24, and 30 did not show evidence of EMT; these patients progressed after 2.0 ± 1.0 cycles (range: 1–3 cycles). The durations of these treatment courses were not significantly different (*P* = 0.68 by unpaired, two-tailed t-test). Only 7 of 31 evaluable patients remained on study for 5 or more cycles and none of these patients were evaluated for EMT.

Two patients with ASPS had the longest courses on study at 22 cycles (patient 2 at DL1) and 18 cycles (patient 9, 3 cycles at DL3 followed by 15 cycles at DL2). Although ASPS is an indolent disease, both patients had objective evidence of disease progression on cediranib prior to enrolling onto the current study; patient 9 had also taken two additional regimens (ifosfamide + doxorubicin and sunitinib). Prolonged periods of stable disease following progression on other regimens suggest a potential benefit of this treatment combination for patients with ASPS. This trial enrolled patients with many different tumor histologies, limiting analyses of outcomes and pharmacodynamics in any one tumor type.

The question of how a combination of anti-angiogenic agents targeting different signaling pathways would affect EMT, and potentially influence drug resistance, remains unanswered, in part because tivantinib is not an authentic inhibitor of c-MET. Circulating VEGF levels increased across all DLs, possibly contributing to the induction of EMT in 4 out of 7 tumor biopsy pairs [20, 21, 46], while levels of other circulating analytes remained more stable. Intact c-MET decreased in 6 or 7 biopsy pairs, although changes in phospho-MET were only quantifiable in 2 patients. No relationship between EMT status and clinical outcome was identified given the number of cycles completed by these patients. Further exploration of tumor responses to anti-angiogenic therapies via EMT-IFA analysis could provide insight into how to optimize the course of treatment for individual patients.

## Supplementary Information

Below is the link to the electronic supplementary material.

Supplementary file1 (PDF 117 KB)

## Declarations

### Clinical trial registration

This trial is registered at www.clinicaltrials.gov (NCT01468922).

## References

[1] Goel HL, Mercurio AM (2013). VEGF targets the tumour cell. Nat Rev Cancer.

[2] Organ SL, Tsao MS (2011). An overview of the c-MET signaling pathway. Ther Adv Med Oncol.

[3] Yamashita J, Ogawa M, Yamashita S, Nomura K, Kuramoto M, Saishoji T, Shin S (1994). Immunoreactive hepatocyte growth factor is a strong and independent predictor of recurrence and survival in human breast cancer. Cancer Res.

[4] Zhang YW, Su Y, Volpert OV, Vande Woude GF (2003). Hepatocyte growth factor/scatter factor mediates angiogenesis through positive VEGF and negative thrombospondin 1 regulation. Proc Natl Acad Sci U S A.

[5] Eppenberger U, Kueng W, Schlaeppi JM, Roesel JL, Benz C, Mueller H, Matter A, Zuber M, Luescher K, Litschgi M, Schmitt M, Foekens JA, Eppenberger-Castori S (1998). Markers of tumor angiogenesis and proteolysis independently define high- and low-risk subsets of node-negative breast cancer patients. J Clin Oncol.

[6] Linderholm B, Grankvist K, Wilking N, Johansson M, Tavelin B, Henriksson R (2000). Correlation of vascular endothelial growth factor content with recurrences, survival, and first relapse site in primary node-positive breast carcinoma after adjuvant treatment. J Clin Oncol.

[7] Gerritsen ME, Tomlinson JE, Zlot C, Ziman M, Hwang S (2003). Using gene expression profiling to identify the molecular basis of the synergistic actions of hepatocyte growth factor and vascular endothelial growth factor in human endothelial cells. Br J Pharmacol.

[8] Van Belle E, Witzenbichler B, Chen D, Silver M, Chang L, Schwall R, Isner JM (1998). Potentiated Angiogenic Effect of Scatter Factor/Hepatocyte Growth Factor via Induction of Vascular Endothelial Growth Factor : The Case for Paracrine Amplification of Angiogenesis. Circulation.

[9] Xin X, Yang S, Ingle G, Zlot C, Rangell L, Kowalski J, Schwall R, Ferrara N, Gerritsen ME (2001). Hepatocyte Growth Factor Enhances Vascular Endothelial Growth Factor-Induced Angiogenesis in Vitro and in Vivo. Am J Pathol.

[10] Sennino B, Ishiguro-Oonuma T, Wei Y, Naylor RM, Williamson CW, Bhagwandin V, Tabruyn SP, You WK, Chapman HA, Christensen JG, Aftab DT, McDonald DM (2012). Suppression of tumor invasion and metastasis by concurrent inhibition of c-Met and VEGF signaling in pancreatic neuroendocrine tumors. Cancer Discov.

[11] Itatani Y, Kawada K, Yamamoto T, Sakai Y (2018) Resistance to Anti-Angiogenic Therapy in Cancer-Alterations to Anti-VEGF Pathway. Int J Mol Sci 19(4). 10.3390/ijms19041232 10.3390/ijms19041232 PMC5979390 29670046

[12] Lu KV, Chang JP, Parachoniak CA, Pandika MM, Aghi MK, Meyronet D, Isachenko N, Fouse SD, Phillips JJ, Cheresh DA, Park M, Bergers G (2012). VEGF inhibits tumor cell invasion and mesenchymal transition through a MET/VEGFR2 complex. Cancer Cell.

[13] Yap TA, de Bono JS (2010). Targeting the HGF/c-Met Axis: State of Play. Mol Cancer Ther.

[14] Imai T, Horiuchi A, Wang C, Oka K, Ohira S, Nikaido T, Konishi I (2003). Hypoxia attenuates the expression of E-cadherin via up-regulation of SNAIL in ovarian carcinoma cells. Am J Pathol.

[15] Evans AJ, Russell RC, Roche O, Burry TN, Fish JE, Chow VW, Kim WY, Saravanan A, Maynard MA, Gervais ML, Sufan RI, Roberts AM, Wilson LA, Betten M, Vandewalle C, Berx G, Marsden PA, Irwin MS, Teh BT, Jewett MA, Ohh M (2007). VHL promotes E2 box-dependent E-cadherin transcription by HIF-mediated regulation of SIP1 and snail. Mol Cell Biol.

[16] Mak P, Leav I, Pursell B, Bae D, Yang X, Taglienti CA, Gouvin LM, Sharma VM, Mercurio AM (2010). ERbeta impedes prostate cancer EMT by destabilizing HIF-1alpha and inhibiting VEGF-mediated snail nuclear localization: implications for Gleason grading. Cancer Cell.

[17] Krishnamachary B, Zagzag D, Nagasawa H, Rainey K, Okuyama H, Baek JH, Semenza GL (2006). Hypoxia-inducible factor-1-dependent repression of E-cadherin in von Hippel-Lindau tumor suppressor-null renal cell carcinoma mediated by TCF3, ZFHX1A, and ZFHX1B. Cancer Res.

[18] Erler JT, Bennewith KL, Nicolau M, Dornhofer N, Kong C, Le QT, Chi JT, Jeffrey SS, Giaccia AJ (2006). Lysyl oxidase is essential for hypoxia-induced metastasis. Nature.

[19] Yang MH, Wu MZ, Chiou SH, Chen PM, Chang SY, Liu CJ, Teng SC, Wu KJ (2008). Direct regulation of TWIST by HIF-1alpha promotes metastasis. Nat Cell Biol.

[20] Gonzalez-Moreno O, Lecanda J, Green JE, Segura V, Catena R, Serrano D, Calvo A (2010). VEGF elicits epithelial-mesenchymal transition (EMT) in prostate intraepithelial neoplasia (PIN)-like cells via an autocrine loop. Exp Cell Res.

[21] Yang AD, Camp ER, Fan F, Shen L, Gray MJ, Liu W, Somcio R, Bauer TW, Wu Y, Hicklin DJ, Ellis LM (2006). Vascular endothelial growth factor receptor-1 activation mediates epithelial to mesenchymal transition in human pancreatic carcinoma cells. Cancer Res.

[22] Batchelor TT, Sorensen AG, di Tomaso E, Zhang WT, Duda DG, Cohen KS, Kozak KR, Cahill DP, Chen PJ, Zhu M, Ancukiewicz M, Mrugala MM, Plotkin S, Drappatz J, Louis DN, Ivy P, Scadden DT, Benner T, Loeffler JS, Wen PY, Jain RK (2007). AZD2171, a pan-VEGF receptor tyrosine kinase inhibitor, normalizes tumor vasculature and alleviates edema in glioblastoma patients. Cancer Cell.

[23] Kumar R, Knick VB, Rudolph SK, Johnson JH, Crosby RM, Crouthamel MC, Hopper TM, Miller CG, Harrington LE, Onori JA, Mullin RJ, Gilmer TM, Truesdale AT, Epperly AH, Boloor A, Stafford JA, Luttrell DK, Cheung M (2007). Pharmacokinetic-pharmacodynamic correlation from mouse to human with pazopanib, a multikinase angiogenesis inhibitor with potent antitumor and antiangiogenic activity. Mol Cancer Ther.

[24] Podar K, Tonon G, Sattler M, Tai Y-T, LeGouill S, Yasui H, Ishitsuka K, Kumar S, Kumar R, Pandite LN, Hideshima T, Chauhan D, Anderson KC (2006). The small-molecule VEGF receptor inhibitor pazopanib (GW786034B) targets both tumor and endothelial cells in multiple myeloma. Proc Natl Acad Sci.

[25] Sonpavde G, Hutson TE, Sternberg CN (2008). Pazopanib, a potent orally administered small-molecule multitargeted tyrosine kinase inhibitor for renal cell carcinoma. Expert Opin Investig Drugs.

[26] de Wit D, van Erp NP, den Hartigh J, Wolterbeek R, den Hollander-van DM, Labots M, Guchelaar HJ, Verheul HM, Gelderblom H (2015). Therapeutic drug monitoring to individualize the dosing of pazopanib: a pharmacokinetic feasibility study. Ther Drug Monit.

[27] D'Cunha R, Bae S, Murry DJ, An G (2016). TKI combination therapy: strategy to enhance dasatinib uptake by inhibiting Pgp- and BCRP-mediated efflux. Biopharm Drug Dispos.

[28] Sauzay C, White-Koning M, Hennebelle I, Deluche T, Delmas C, Imbs DC, Chatelut E, Thomas F (2016) Inhibition of OCT2, MATE1 and MATE2-K as a possible mechanism of drug interaction between pazopanib and cisplatin. Pharmacol Res 11089–95. 10.1016/j.phrs.2016.05.012 10.1016/j.phrs.2016.05.012 27178732

[29] Filppula AM, Neuvonen PJ, Backman JT (2014). In vitro assessment of time-dependent inhibitory effects on CYP2C8 and CYP3A activity by fourteen protein kinase inhibitors. Drug Metab Dispos.

[30] Xu CF, Bing NX, Ball HA, Rajagopalan D, Sternberg CN, Hutson TE, de Souza P, Xue ZG, McCann L, King KS, Ragone LJ, Whittaker JC, Spraggs CF, Cardon LR, Mooser VE, Pandite LN (2011). Pazopanib efficacy in renal cell carcinoma: evidence for predictive genetic markers in angiogenesis-related and exposure-related genes. J Clin Oncol.

[31] Basilico C, Pennacchietti S, Vigna E, Chiriaco C, Arena S, Bardelli A, Valdembri D, Serini G, Michieli P (2013). Tivantinib (ARQ197) displays cytotoxic activity that is independent of its ability to bind MET. Clin Cancer Res.

[32] Katayama R, Aoyama A, Yamori T, Qi J, Oh-hara T, Song Y, Engelman JA, Fujita N (2013). Cytotoxic activity of tivantinib (ARQ 197) is not due solely to c-MET inhibition. Cancer Res.

[33] Yamamoto N, Murakami H, Nishina T, Hirashima T, Sugio K, Muro K, Takahashi T, Naito T, Yasui H, Akinaga S, Koh Y, Boku N (2013). The effect of CYP2C19 polymorphism on the safety, tolerability, and pharmacokinetics of tivantinib (ARQ 197): results from a phase I trial in advanced solid tumors. Ann Oncol.

[34] Matsumoto K, Umitsu M, De Silva DM, Roy A, Bottaro DP (2017). Hepatocyte growth factor/MET in cancer progression and biomarker discovery. Cancer Sci.

[35] Srivastava AK, Hollingshead MG, Weiner J, Navas T, Evrard YA, Khin SA, Ji JJ, Zhang Y, Borgel S, Pfister TD, Kinders RJ, Bottaro DP, Linehan WM, Tomaszewski JE, Doroshow JH, Parchment RE (2016). Pharmacodynamic Response of the MET/HGF Receptor to Small-Molecule Tyrosine Kinase Inhibitors Examined with Validated. Fit-for-Clinic Immunoassays Clin Cancer Res.

[36] Navas T, Srivastava AK, Govindharajulu JP, Evrard YA, Borgel S, Carter J, Chen L, Das B, Divelbiss R, Karlovich C, Patidar R, Radzyminski M, Stottlemyer J, Williams PM, Hollingshead MG, Bottaro D, Doroshow JH, Parchment RE (2019) Measuring phospho-MET by multiplex immunofluorescence to aid in selection of patients with MET activation in tumors. J Clin Oncol. 37(15_suppl):3131-. 10.1200/JCO.2019.37.15_suppl.3131

[37] Jun HJ, Lee J, Lim DH, Park JO, Ahn G, Seo SW, Sung KS, Lim DH, Yoo KH, Choi YL (2010). Expression of MET in alveolar soft part sarcoma. Med Oncol.

[38] Heath EI, Chiorean EG, Sweeney CJ, Hodge JP, Lager JJ, Forman K, Malburg L, Arumugham T, Dar MM, Suttle AB, Gainer SD, LoRusso P (2010). A phase I study of the pharmacokinetic and safety profiles of oral pazopanib with a high-fat or low-fat meal in patients with advanced solid tumors. Clin Pharmacol Ther.

[39] Imbs DC, Paludetto MN, Negrier S, Powell H, Lafont T, White-Koning M, Chatelut E, Thomas F (2016). Determination of unbound fraction of pazopanib in vitro and in cancer patients reveals albumin as the main binding site. Invest New Drugs.

[40] Tan AR, Gibbon DG, Stein MN, Lindquist D, Edenfield JW, Martin JC, Gregory C, Suttle AB, Tada H, Botbyl J, Stephenson JJ (2013). Effects of ketoconazole and esomeprazole on the pharmacokinetics of pazopanib in patients with solid tumors. Cancer Chemother Pharmacol.

[41] D Goldstein JE Rosenberg RA Figlin RR Townsend L McCann C Carpenter L Pandite 2016 Is change in blood pressure a biomarker of pazopanib and sunitinib efficacy in advanced/metastatic renal cell carcinoma? Eur J Cancer 5396–104. 10.1016/j.ejca.2015.10.006 10.1016/j.ejca.2015.10.006 26702763

[42] Suttle AB, Ball HA, Molimard M, Hutson TE, Carpenter C, Rajagopalan D, Lin Y, Swann S, Amado R, Pandite L (2014). Relationships between pazopanib exposure and clinical safety and efficacy in patients with advanced renal cell carcinoma. Br J Cancer.

[43] Harris PA, Boloor A, Cheung M, Kumar R, Crosby RM, Davis-Ward RG, Epperly AH, Hinkle KW, Hunter RN, Johnson JH, Knick VB, Laudeman CP, Luttrell DK, Mook RA, Nolte RT, Rudolph SK, Szewczyk JR, Truesdale AT, Veal JM, Wang L, Stafford JA (2008). Discovery of 5-[[4-[(2,3-dimethyl-2H-indazol-6-yl)methylamino]-2-pyrimidinyl]amino]-2-methyl-b enzenesulfonamide (Pazopanib), a novel and potent vascular endothelial growth factor receptor inhibitor. J Med Chem.

[44] Peach CJ, Mignone VW, Arruda MA, Alcobia DC, Hill SJ, Kilpatrick LE, Woolard J (2018) Molecular Pharmacology of VEGF-A Isoforms: Binding and Signalling at VEGFR2. Int J Mol Sci 19(4). 10.3390/ijms19041264 10.3390/ijms19041264 PMC5979509 29690653

[45] Pennacchietti S, Michieli P, Galluzzo M, Mazzone M, Giordano S, Comoglio PM (2003). Hypoxia promotes invasive growth by transcriptional activation of the met protooncogene. Cancer Cell.

[46] Navas T, Kinders RJ, Lawrence SM, Ferry-Galow KV, Borgel S, Hollingshead MG, Srivastava AK, Alcoser SY, Makhlouf HR, Chuaqui R, Wilsker DF, Konate MM, Miller SB, Voth AR, Chen L, Vilimas T, Subramanian J, Rubinstein L, Kummar S, Chen AP, Bottaro DP, Doroshow JH, Parchment RE (2020). Clinical Evolution of Epithelial-Mesenchymal Transition in Human Carcinomas. Cancer Res.

