# Supplementary material for: Combination therapy with pazopanib and tivantinib modulates VEGF and c-MET levels in refractory advanced solid tumors
Source: Invest New Drugs. 2021 Jun 28;39(6):1577–86. doi: 10.1007/s10637-021-01138-x (PMC8541958; doi:10.1007/s10637-021-01138-x)
Supplement: Supplementary file 1 — Supplementary file1 (PDF 117 KB) [file 10637_2021_1138_MOESM1_ESM.pdf]

**Supplementary Materials**

Supplementary Table S1: Patient demographics

| | |
|---|---|
| Patients enrolled | 32 |
| Patients evaluable | 31 |
| Mean age (years) | 49 |
| Median age (years) | 53.5 |
| Age range (years) | 20-73 |
| | |
| Gender | |
| Female | 14 |
| Male | 18 |
| | |
| Race | |
| Caucasian | 20 |
| Asian | 5 |
| African American | 3 |
| Unknown | 3 |
| American Indian or Alaska Native | 1 |
| | |
| Accrual by Dose Level | |
| DL1 | 3 |
| DL2 | 3 |
| DL3 | 3 |
| DL4 | 6 |
| DL5 | 17 |

Supplementary Table S2: Adverse events by dose level.

| Adverse Event | Grade | DL1 (n=3) | DL2 (n=3) | DL3 (n=3) | DL4 (n=6) | DL5 (n=16) |
|---|---|---|---|---|---|---|
| Alanine aminotransferase increased | 2 | | | | | 1 |
| | 3 | | | | 2 | |
| Alkaline phosphatase increased | 2 | | 1 | | | 1 |
| Allergic reaction | 2 | | | | 1 | |
| Alopecia | 2 | | | | 1 | |
| Anemia | 2 | | | | | 1 |
| | 3 | | | | | 2 |
| Anorexia | 2 | | 1 | | | 1 |
| Aspartate aminotransferase increased | 3 | | | | 2 | |
| Creatine increased | 2 | | | | | 1 |
| Dehydration | 2 | | 1 | | | |
| Dry skin | 2 | | | | | 1 |
| Dysgeusia | 2 | | | | | 1 |
| Fatigue | 2 | | 1 | | | |
| Febrile neutropenia | 4 | | | | | 2 |
| Flatulence | 2 | | 1 | | | |
| Hypertension | 2 | 3 | 2 | 2 | 3 | 5 |
| | 3 | | | | 1 | 2 |
| Hypoalbuminemia | 2 | | 1 | | | 1 |
| Hyperbilirubinemia | 2 | | | | | 3 |
| Hypophosphatemia | 2 | | | 1 | | 1 |
| Leucopenia | 2 | 1 | | | 1 | 4 |
| | 3 | | | | 2 | |
| | 4 | | | | | 2 |
| Lower gastrointestinal hemorrhage | 2 | | | | | 1 |
| Lymphopenia | 2 | 1 | | | 2 | |
| | 3 | 1 | | | 1 | 2 |
| | 4 | | | | | 1 |
| Nausea | 2 | | 1 | | | 1 |
| Neutropenia | 2 | 1 | | | | 3 |
| | 3 | | | | 1 | 1 |
| | 4 | | | | | 1 |
| Sepsis | 4 | | | | | 2 |
| Skin Hypopigmentation | 2 | | | | | 1 |
| Stomach Pain | 2 | 1 | | | | |
| Thrombocytopenia | 2 | | | | | 2 |
| Weight loss | 2 | | 1 | | | |

Supplementary Table S3: Histologies by patient

| Patient | Histology |
|---|---|
| 1 | Synovial sarcoma |
| 2 | Alveolar soft part sarcoma |
| 3 | Hurthle cell thyroid carcinoma |
| 4 | Metastatic colon adenocarcinoma |
| 5 | Hemangiopericytoma |
| 6 | Pleural mesothelioma |
| 7 | Chondrosarcoma |
| 8 | Malignant mesothelioma |
| 9 | Alveolar soft part sarcoma |
| 10 | Myxoid liposarcoma |
| 11 | Adenocarcinoma |
| 12 | Adenocarcinoma |
| 13 | Hepatocellular carcinoma |
| 14 | Granular cell tumor |
| 15 | Alveolar rhabdomyosarcoma |
| 16 | Adenocystic carcinoma |
| 17 | Metastatic renal cell carcinoma |
| 18 | Papillary serous adenocarcinoma - ovary |
| 19 | Non-small cell lung carcinoma |
| 20 | Prostatic adenocarcinoma |
| 21 | Neuroendocrine tumor |
| 22 | Metastatic granulosa cell tumor |
| 23 | Malignant chondrosarcoma |
| 24 | Myoepithelial adenocarcinoma - parotid gland |
| 25 | Renal cell carcinoma |
| 26 | Unspecified carcinoma |
| 27 | Leiomyosarcoma |
| 28 | Ovarian carcinoma |
| 29 | Non-small cell lung carcinoma |
| 30 | Esophageal carcinoma |
| 31 | Colonic adenocarcinoma |
| 32 | Metastatic colonic adenocarcinoma |

Supplementary Table S4: Pazopanib PK values by patient.

| Pazopanib Dose (mg) | Dose Level | Patient | **Day 1** $C_{max}$ (ng/mL) | $AUC_{last}$ (h*ng/mL) | $T_{max}$ (h) | **Day 15** $C_{max}$ (ng/mL) | $AUC_{last}$ (h*ng/mL) | $T_{max}$ (h) | D15/D1 AUC Ratio |
|---|---|---|---|---|---|---|---|---|---|
| 600 | 2 | 5 | 33 | 301 | 12 | 75 | 369 | 4 | 1.23 |
| (n=2) | | 6 | 10 | 83 | 1 | 20 | 109 | 2 | 1.31 |
| | | Mean | 22 | 192 | 6.5 | 48 | 239 | 3.0 | 1.27 |
| | | SD | 16 | 154 | 7.8 | 39 | 184 | 1.4 | 0.06 |
| 600 | 3 | 7 | 33 | 300 | 4 | 40 | 223 | 4 | 0.74 |
| (D1 n=3, | | 8 | 28 | 244 | 4 | 18 | 100 | 2 | 0.41 |
| D15 n=2) | | 9 | 36 | 310 | 4 | | | | |
| | | Mean | 32 | 285 | 4.0 | 29 | 162 | 3.0 | 0.58 |
| | | SD | 4 | 36 | 0.0 | 16 | 87 | 1.4 | 0.24 |
| 800 | 4 | 10 | 19 | 150 | 2 | 57 | 297 | 4 | 1.98 |
| (D1 n=6, | | 11 | 64 | 566 | 4 | 90 | 437 | 4 | 0.77 |
| D15 n=5) | | 12 | 39 | 312 | 4 | 46 | 220 | 4 | 0.71 |
| | | 13 | 100 | 735 | 2 | 21 | 91 | 6 | 0.12 |
| | | 14 | 39 | 347 | 4 | 100 | 463 | 4 | 1.33 |
| | | 15 | 24 | 229 | 4 | | | | |
| | | Mean | 48 | 390 | 3.3 | 63 | 302 | 4.4 | 0.98 |
| | | SD | 30 | 220 | 1.0 | 32 | 154 | 0.9 | 0.70 |
| 800 | 5 | 16 | 23 | 197 | 2 | 58 | 29 | 1 | 0.15 |
| (n=6) | | 18 | 92 | 796 | 6 | 71 | 337 | 4 | 0.42 |
| | | 19 | 64 | 559 | 4 | 59 | 266 | 6 | 0.48 |
| | | 20 | 4 | 30 | 1 | 36 | 120 | 6 | 4.00 |
| | | 21 | 16 | 125 | 2 | 20 | 97 | 1 | 0.78 |
| | | 22 | 13 | 97 | 2 | 75 | 334 | 4 | 3.44 |
| | | Mean | 35 | 301 | 2.8 | 53 | 197 | 3.7 | 1.54 |
| | | SD | 35 | 306 | 1.8 | 21 | 132 | 2.3 | 1.71 |

Supplementary Table S5: Tivantinib PK values by patient.

| Tivantinib Dose (mg) | Dose Level | Patient | Day 1 $C_{max}$ (ng/mL) | Day 1 $AUC_{last}$ (h*ng/mL) | Day 1 $T_{max}$ (h) | Day 15 $C_{max}$ (ng/mL) | Day 15 $AUC_{last}$ (h*ng/mL) | Day 15 $T_{max}$ (h) | D15/D1 AUC Ratio |
|---|---|---|---|---|---|---|---|---|---|
| 120 | 1 | 1 | 365 | 2540 | 6 | 227 | 830 | 2 | 0.33 |
| (n=3) | | 2 | 414 | 2649 | 4 | 305 | 1268 | 6 | 0.48 |
| | | 3 | 1840 | 15560 | 4 | 2030 | 9685 | 4 | 0.62 |
| | | Mean | 873 | 6916 | 4.7 | 854 | 3928 | 4.0 | 0.48 |
| | | SD | 838 | 7486 | 1.2 | 1019 | 4991 | 2.0 | 0.15 |
| 120 | 2 | 5 | 1760 | 12536 | 4 | 1120 | 4221 | 4 | 0.34 |
| (n=2) | | 6 | 407 | 3037 | 2 | 821 | 3411 | 2 | 1.12 |
| | | Mean | 1084 | 7787 | 3.0 | 971 | 3816 | 3.0 | 0.73 |
| | | SD | 957 | 6717 | 1.4 | 211 | 573 | 1.4 | 0.56 |
| 240 | 3 | 7 | 609 | 4557 | 6 | 495 | 1777 | 4 | 0.39 |
| (D1 n=3, | | 8 | 2860 | 25080 | 6 | 3710 | 20790 | 4 | 0.83 |
| D15 n=2) | | 9 | 1520 | 10850 | 6 | | | | |
| | | Mean | 1663 | 13496 | 6.0 | 2103 | 11284 | 4.0 | 0.61 |
| | | SD | 1132 | 10514 | 0.0 | 2273 | 13444 | 0.0 | 0.31 |
| 240 | 4 | 10 | 1990 | 10583 | 4 | 1200 | 4775 | 4 | 0.45 |
| (n=5) | | 11 | 1910 | 10894 | 4 | 1230 | 3545 | 4 | 0.33 |
| | | 12 | 969 | 6256 | 4 | 773 | 3082 | 2 | 0.49 |
| | | 13 | 2940 | 26410 | 6 | 5470 | 28530 | 2 | 1.08 |
| | | 14 | 804 | 6017 | 6 | 642 | 2717 | 4 | 0.45 |
| | | Mean | 1723 | 12032 | 4.8 | 1863 | 8530 | 3.2 | 0.56 |
| | | SD | 866 | 8362 | 1.1 | 2033 | 11207 | 1.1 | 0.30 |
| 360 | 5 | 16 | 1570 | 12029 | 6 | 800 | 400 | 1 | 0.03 |
| (n=6) | | 18 | 2730 | 18914 | 6 | 1390 | 4799 | 6 | 0.25 |
| | | 19 | 2740 | 22680 | 6 | 1430 | 5019 | 6 | 0.22 |
| | | 20 | 966 | 6235 | 4 | 753 | 3210 | 4 | 0.51 |
| | | 21 | 316 | 1471 | 2 | 473 | 1971 | 2 | 1.34 |
| | | 22 | 1210 | 7655 | 6 | 755 | 3096 | 4 | 0.40 |
| | | Mean | 1589 | 11497 | 5.0 | 934 | 3083 | 3.8 | 0.46 |
| | | SD | 978 | 8041 | 1.7 | 387 | 1740 | 2.0 | 0.46 |
